# Nutrient Contents of the Freshwater Crab, *Isolapotamon bauense* from Sarawak, Malaysia (Borneo)

**DOI:** 10.21315/tlsr2017.28.2.6

**Published:** 2017-07-31

**Authors:** Jongkar Grinang, Pang Sing Tyan, Andrew Alek Tuen, Indraneil Das

**Affiliations:** Institute of Biodiversity and Environmental Conservation, Universiti Malaysia Sarawak, 94300 Kota Samarahan, Sarawak

**Keywords:** Nutrient Contents, Freshwater Crab, *Isolapotamon bauense*, Borneo

## Abstract

Data on nutrient contents of freshwater crabs are important for ecological studies and species conservation assessments, especially when the species concerned is threatened among others by habitat destruction and uncontrolled resources utilisation. Indeed comprehensive biological information is required to reconcile the needs between sustainable resources utilisation and conservation of the species. This study documents the nutrient contents of a freshwater crab, *Isolapotamon bauense* which is listed as ‘Vulnerable’ in the IUCN Red List of Threatened Species and also being harvested by local community for dietary supplement. Results show that muscles of the freshwater crab contain a substantial amount of nutrients in particular water content (male = 79.31 ± 2.30 %, female = 77.63 ± 0.56 %), protein (male = 77.47 ± 6.11 %, female = 63.28 ± 3.62 %), magnesium (male = 51.48 ± 16.10 mg/g, female = 39.73 ± 6.99 mg/g) and calcium (male = 25.50 ± 6.98 mg/g, female = 39.73 ± 6.99 mg/g). Means of nutrient contents between male and female crabs are not significantly different. It is estimated that an individual of *I. bauense* with weight range of 56–139 g contained on average of 0.35 ± 0.15 g of protein. Our estimation also shows that the number of individuals of the freshwater crab required to meet the recommended daily protein intakes of the community concerned is in the range 35–96 individuals for children, 130–188 individuals for adolescents, 171–179 individuals for men and 149–159 individuals for women. The results imply that harvesting of wild *I. bauense* as a source of protein supplement naturally may not be practical because of its relatively low population abundance, and conservation of the species for its ecological roles may thus be preferred.

## Abstract (ru)

Data mengenai kandungan nutrien ketam air tawar adalah penting untuk kajian ekologi dan penilaian pemuliharaan spesies, terutamanya apabila spesies yang berkenaan terancam, antara lain oleh pemusnahan habitat dan penggunaan sumber yang tidak terkawal. Maklumat biologi yang komprehensif diperlukan untuk menyelaraskan keperluan antara pemanfaatan sumber pemuliharaan dan pemuliharaan spesies. Kajian ini menyenaraikan kandungan nutrien ketam air tawar, *Isolapotamon bauense* yang disenaraikan sebagai ‘Kerentanan’ dalam Senarai Merah Spesies Terancam IUCN dan juga ditangkap oleh masyarakat tempatan untuk makanan tambahan. Keputusan menunjukkan bahawa otot ketam air tawar mengandungi sejumlah besar nutrien dalam kandungan air tertentu (jantan = 79.31 ± 2.30%, betina = 77.63 ± 0.56%), protein (jantan = 77.47 ± 6.11%, betina = 63.28 ± 3.62%), Magnesium (jantan = 51.48 ± 16.10 mg/g, betina = 39.73 ± 6.99 mg/g) dan kalsium (jantan = 25.50 ± 6.98 mg/g, betina = 39.73 ± 6.99 mg/g). Kandungan nutrien antara ketam jantan dan betina tidak begitu ketara. Adalah dianggarkan bahawa seekor ketam *I. bauense* dengan julat berat 56–139 g yang mengandungi purata 0.35 ± 0.15 g protein. Pengiraan juga menunjukkan bahawa bilangan ketam air tawar yang diperlukan untuk memenuhi pengambilan protein harian yang disyorkan bagi masyarakat berkenaan adalah dalam lingkungan 35–96 ekor untuk kanak-kanak, 130–188 ekor untuk remaja, 171–179 ekor untuk lelaki dewasa dan 149–159 ekor untuk wanita dewasa. Hasilnya menandakan bahawa penangkapan *I. bauense* liar sebagai sumber suplemen protein semulajadi mungkin tidak praktikal kerana kelimpahan penduduknya yang agak rendah, dan pemuliharaan spesies untuk peranan ekologinya boleh diutamakan.

## INTRODUCTION

Analyses of nutrient contents of freshwater crabs are fundamental for both ecological studies and species conservation assessments. They are useful in understanding predator-prey relationships, nutrient cycles, and food chains in the freshwater ecosystem. Such ecological knowledge, however, is lacking in the tropics, although studies have shown freshwater crabs are the primary food sources for many aquatic and terrestrial animals. In the Upper Kairezi River of Zimbabwe, the African freshwater crab, *Potamonautes perlatus* is a major item in the diet of the Cape clawless otter (*Aonyx capensis*), African mottled eels (*Anguilla labiata*) and rainbow trout (*Oncorhynchus mykiss*) (Turnbull-Kemp 1960; Butler & Marshal 1996). The importance of crabs as food for the Cape clawless otter was also demonstrated in Bloukrans River, Eastern Cape Province of South Africa (Parker *et al*. 2005). Other animals that prey on freshwater crabs include reptiles and amphibians (Ng 1988, 1995; Jensen & Das 2006). Also, freshwater crabs are among the most common shredders and significantly contribute to nutrient cycling in many tropical streams (Hill & O’Keeffe 1992; Covich & McDowell 1996; Dobson *et al*. 2007; Marijnissen *et al*. 2008; Yule *et al*. 2009) as well as in litter degradation within alluvial forests (Collins 1980).

Nutritional analyses also have the potential to resolve conflicts between utilisation of freshwater crab resource by indigenous human communities and species conservation programmes. The importance of freshwater crabs as the source of food for indigenous communities in Asian countries has been mentioned by some authors (e.g., Ng 1988; Yeo & Ng 1998; Cumberlidge *et al*. 2009; Mendoza & Naruse 2010; Ghosh-Jerath *et al*. 2015). Such communities consume freshwater crabs as a source of protein, and additionally, for medicinal reasons: ashes from burnt crabs are used for the treatment of tuberculosis, jaundice, liver disorder, cough, asthma, and injuries (Jamir & Lal 2005; Mahawar & Jaroli 2007; Yeo *et al*. 2008; Banerjee *et al*. 2010). Consumption of freshwater crabs, mainly as a dietary supplement, has been reported in Sarawak (Grinang, 2016). While utilization of freshwater crabs has been broadly documented, information on nutrient contents of these resources remain scarce (but see Adeyeye 2002; Bilgin & Fidanbaş 2011; Omotayo *et al*. 2013: Sudha Devi & Smija 2013; Varadharajan & Soundarapandian 2014).

Research on freshwater crabs in Sarawak started in the early of 1900s, and the current list of decapod crustacean comprises three families, 14 genera and 48 species (Ng *et al*. 2008; Grinang 2016). Of these, four species are listed as ‘Endangered’ and two species are ‘Vulnerable’ in the IUCN Red List of Threatened Species (IUCN 2015). An additional 42 species are categorised either as ‘Least Concern’, ‘Data Deficient’, or being unassessed. Conservation assessment of freshwater crabs in the region has progressed slowly, due to insufficient information on ecology and threats to species (see Ng & Yeo 2007; McFarlane & Lundberg 2012). For instance, preliminary conservation assessment of *Isolapotamon bauense* has classified it as ‘Vulnerable’ due to its population size and threats not being known (Ng & Yeo 2007; Cumberlidge *et al*. 2009; IUCN 2015). Presently, certain ecological characteristics of the species, including distributional range, population structure, growth pattern, condition and population size have been published (Grinang *et al*. 2016), and utilisation of the resource by indigenous communities has also been reported (Grinang 2016). Mohd-Azlan and Fauzi (2006) noted that wildlife harvesting by Iban, Bidayuh, Malays and Melanau in Sarawak are associated with daily livelihoods of the communities specifically the cultures, beliefs and traditional farming practices. In Gunung Serambu, anecdotal evident indicates that *I. bauense* (locally known as *Kuyoh Gunong,* literally ‘montane crab’) along with other aquatic life include fishes, molluscs and frogs are harvested by the Bidayuh community primarily as food supplements, rather than for other purposes. The primary objective of this study is to document the nutrient contents of *I. bauense*, which will be useful information, complementing the conservation assessment of the species in the future. A better understanding of the nutritional value of their food by indigenous communities and its availability may enhance their awareness of the need for sustainable utilisation and conservation.

## MATERIALS AND METHODS

### Study Sites

**Figure 1:**
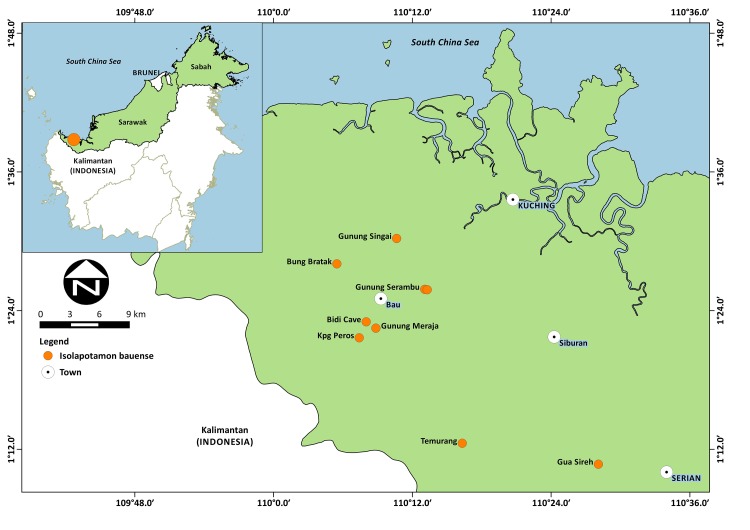
Map showing the study site at Gunung Serambu in Bau District, Sarawak, East Malaysia (Borneo), and the known distribution range of *Isolapotamon bauense*.

*Isolapotamon bauense* was described from the limestone cave of Gua Sireh in Serian District, in western Sarawak (Ng 1987), and has only been recorded from isolated localities within Kuching Division (Grinang *et al*. 2016). A collection of *I. bauense* was conducted at Gunung Serambu at Kampung Peninjau Lama (village) in Bau District (Fig. 1). The mountain is a historical site, being associated with Rajah James Brooke’s (1803–1868) cottage, where the celebrated British naturalist, Alfred Russel Wallace (1823–1913) spent time during his explorations in Sarawak. Crabs were collected from two sites at an intermittent stream located to the northeast of the mountain. The stream channel is steep, consisting predominantly rocky outcrop, with massive boulders lining the passages, and the vegetation comprised mixed dipterocarp forest. The stream is ephemeral, flowing after periods of heavy rainfall, although there is the year-round availability of ground water. Site 1 is located on the lower reaches of the intermittent stream (01°25′55″N, 110°13′27″E; 199 m above sea level) and Site 2 is in the upper reaches (01°25′50″N, 110°13′04″E), located 340 m above sea level, and about 100 m south of the Brooke’s cottage site. Collection was carried out at night by hands and using scoop nets. A total of 16 specimens of the crab (three males, 13 females) were caught during five sampling trips (18 and 24 September 2013, 8 and 22 October 2013, 20 March 2014).

### Determination of Nutrient Contents

Determination of nutrient contents of *I. bauense* was similar to standard protocols that had been applied for other crab species (Skonberg & Perkins 2002; Gökoðlu & Yerlikaya 2003; Küçükgülmez *et al*. 2006; Bilgin & Fidanbaş 2011; Moronkola *et al*. 2011). In this exploratory study, the nutritional analysis was performed for selected nutrients such as water content, ash, crude protein and nine minerals (i.e., calcium, magnesium, potassium, sodium, zinc, phosphorus, iron, lead, and copper). Each individual of crab was analysed separately for 12 nutrient contents to examine linear relationships between the nutrient contents and morphological characters of the crab (i.e., carapace width, carapace length, and weight).

Fresh crab specimens were boiled for 15 min to extract the muscles from the carapace. The muscles were then dried at 80°C for 48 h to obtain a constant weight for determining the water content. A known weight of the dried ground muscle was incinerated at 500°C for 5 h for ash determination. Crude protein was determined from the dry samples according to Kjeldahl method, which involves digestion, distillation and titration processes (AOAC 1995). Analysis of the nine minerals was conducted using Perkins Elmer Atomic Absorption Spectrophotometer. All analyses were performed in triplicate.

### Statistical Analyses

**Figure 2:**
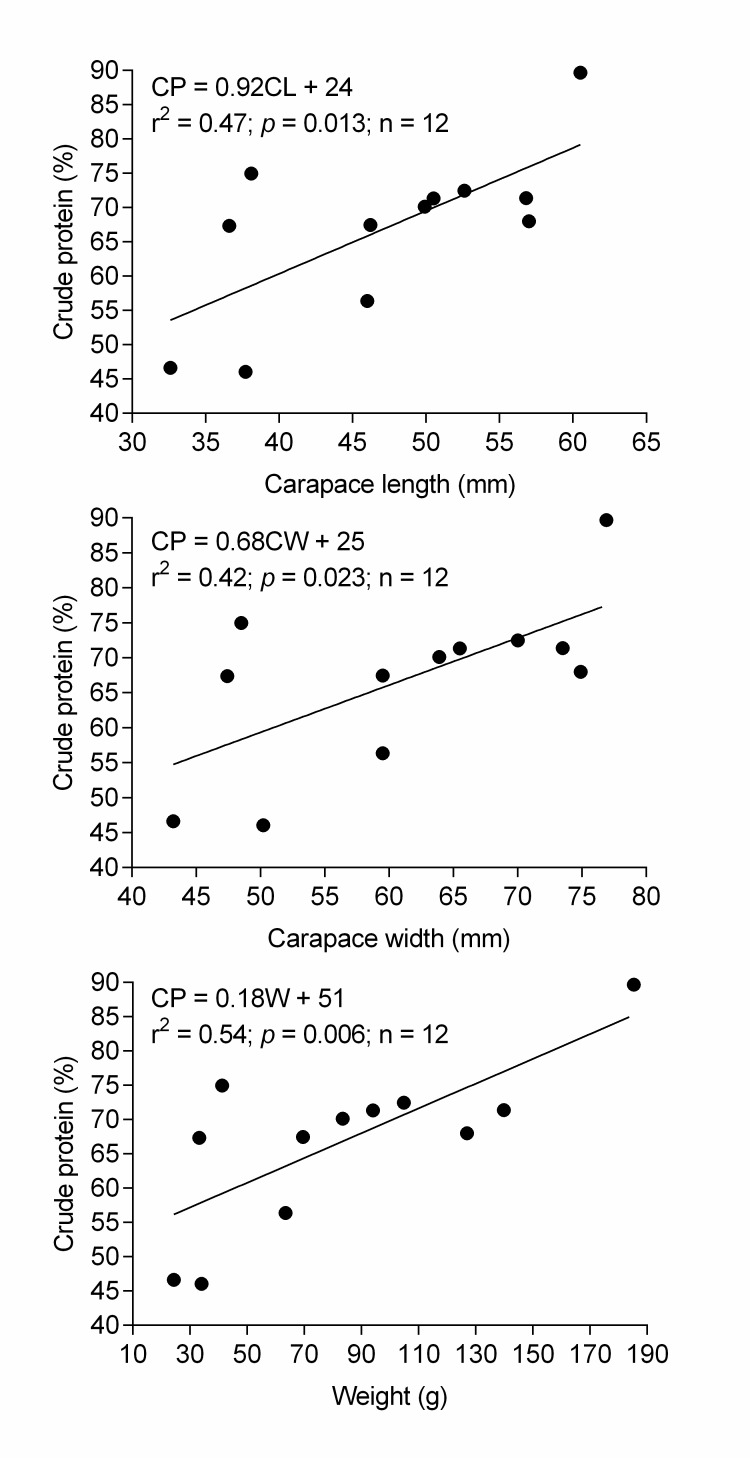
Linear regression between morphological metrics and crude protein of *Isolapotamon bauense*. Pairwise regressions among other metrics were not significant at *p* < 0.05.

The unequal variance of one-tail *t*-test was used to examine if male crabs were significantly larger or heavier than the females. Student *t-*test was performed to test for differences in nutrient contents between sexes of the crab. Microsoft Excel 2013 was used to conduct both analyses. GraphPad Prism Version 6.05 was used to run regression analysis for determining if nutrient contents are dependent on the size or weight of the crab. The number of individuals of *I. bauense* required to meet the recommended daily nutrient intake for indigenous communities was estimated based on Malaysian Dietary Guidelines (NCCFN 2005). The estimation was performed only for crude protein because the value was significantly correlated with size and weight of the crab (Fig. 2). The assumption underlying the estimation is, freshwater crab may constitute a significant item that is daily taken by the indigenous communities.

## RESULTS AND DISCUSSION

**Figure 3:**
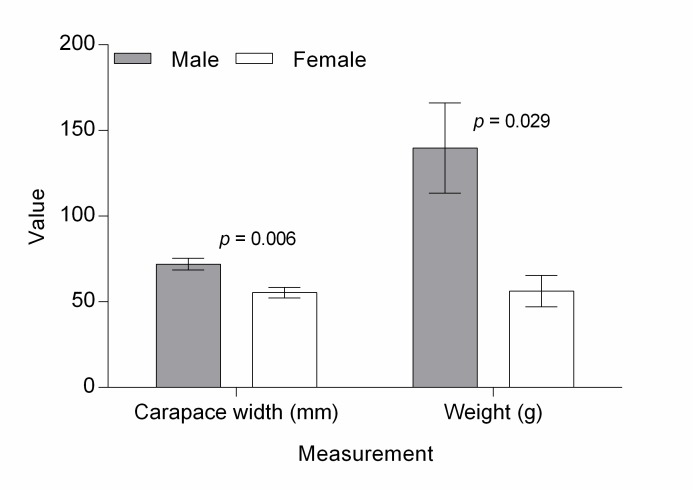
Comparison of carapace width (mm) and weight (g) between male and female of *Isolapotamon bauense*. ♂ = 3, ♀ = 13.

*Isolapotamon bauense* is the largest freshwater crab recorded on Borneo, and can attain a carapace width of 85 mm (Ng 1987). The 16 specimens caught in this study can be grouped as mid-sized, with a range of carapace width of 55–71 mm. The comparative *t*-test showed that male crabs were significantly larger and heavier than female crabs (Figure 3). This large crab is presumed to contain a substantial amount of meat, explaining why it is favoured by the Bidayuh community in Bau District. In Gunung Singai, Grinang (2014) found that the Singai Bidayuh community harvested *I. bauense* occasionally as a source of protein.

**Figure 4:**
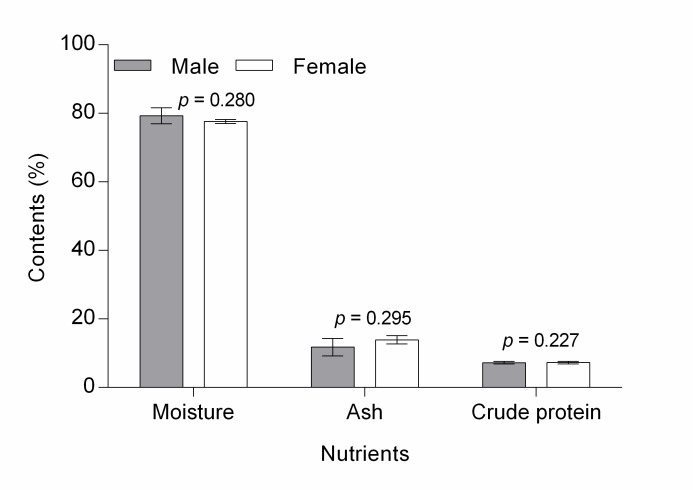
Bar chart shows no significant different of means of moisture, ash and crude protein between male and female of *Isolapotamon bauense* caught from Gunung Serambu, Bau District. ♂ = 3, ♀ = 13.

**Figure 5:**
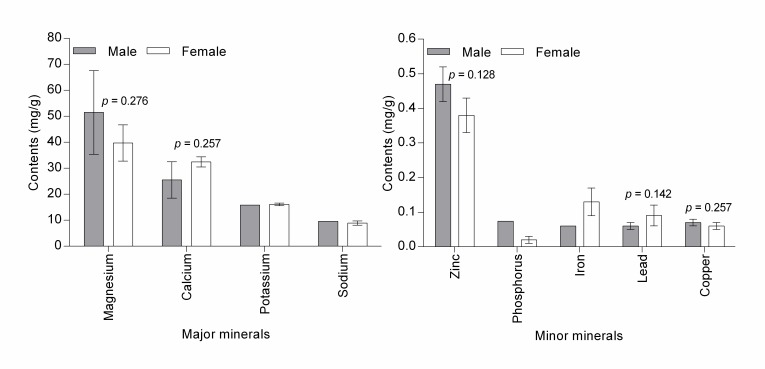
Mineral contents of *Isolapotamon bauense* (mean ± SE) caught from Gunung Serambu, Bau District. No significant different of means of minerals between male and female crabs. Means without standard errors are incomplete analysis due to cross-contamination of the samples. ♂ = 3, ♀ = 13.

The muscles of *I. bauense* have high water content (male = 79.31 ± 2.30%, female = 77.63 ± 0.56%), crude protein (male = 77.47 ± 6.11%, female = 63.28 ± 3.62 %), magnesium (male = 51.48 ± 16.10 mg/g, female = 39.73 ± 6.99 mg/g) and calcium (male = 25.50 ± 6.98 mg/g, female = 39.73 ± 6.99 mg/g) (Fig. 4 and 5). There is no statistical evidence of differences in means of eight nutritional properties between male and female crabs (*p* > 0.05). No comparison was made on means of phosphorus, potassium, sodium and iron between male and female crabs, as samples were cross-contaminated. High water content, crude protein, magnesium, and calcium in *I. bauense* was also demonstrated in other freshwater crabs (Adeyeye 2002; Bilgin & Fidanbaş 2011; Omotayo *et al*. 2014. Sudha Devi & Smija 2013; Varadharajan & Soundarapandian 2014) and marine crabs (Skonberg & Perkins 2002; Gökoðlu & Yerlikaya 2003; Moronkola *et al*. 2011). Nonetheless, there is significant variation in concentrations of the nutrients across studies, which might relate to the preparation of crab muscles before nutrient analyses. Several studies found that proximate composition and mineral analysis are significantly affected by cooking methods (Gökoðlu *et al*. 2004; Ersoy *et al*. 2006; Türkkan *et al*. 2008; Aberoumad 2014). The concentration of nutrients and minerals may be influenced by seasonal and biological differences, food source and the environment itself (Küçükgülmez *et al*. 2006; Bilgin & Fidanbaş 2011). Our findings indicate that only crude protein was significantly dependent on size and weight of the crab (Fig. 2).

**Figure 6:**
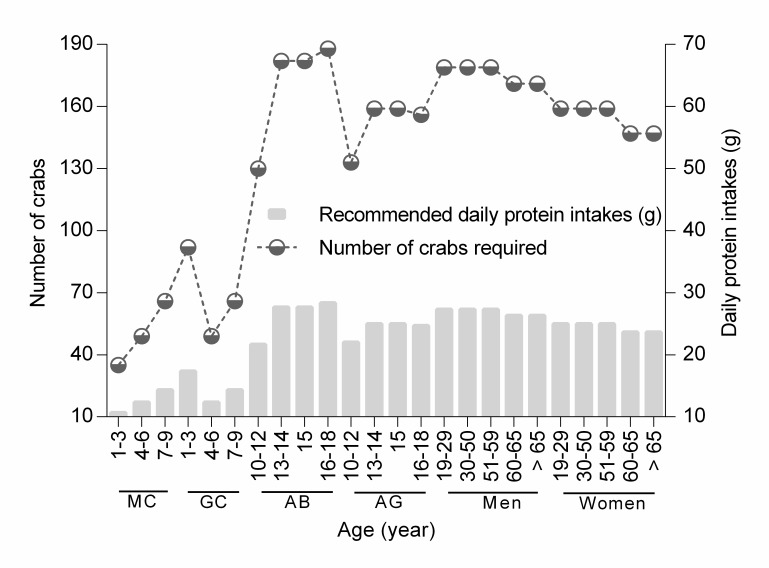
Estimated number of individuals of *Isolapotamon bauense* required to meet the recommended daily protein intakes of indigenous communities. CB = children-boy, CG = children-girl, AB = adolescent boy, AG = adolescent-girl. The estimation is based on the Malaysian Dietary Guideline 2005 (NCCFN, 2005).

Consumption of wild meats (e.g., mammals, reptiles and amphibians) by indigenous communities in Sarawak for dietary supplement had been documented by other authors (Rahman *et al*. 2003; Mohd-Azlan & Fauzi 2006), but information on nutrient properties of the resources is lacking. Our estimation showed that an individual of *I. bauense* (weight range 56–139 g) contained on average of 0.35 ± 0.15 g of crude protein. Based on this average and by assuming that these crabs constituted a major part of the diet of the indigenous community in question, the number of individuals of *I. bauense* required to meet the recommended daily protein intakes of the community is in the range 35–96 individuals for children, 130–188 individuals for adolescents, 171–179 individuals for men and 149–159 individuals for women (Fig. 6). This estimation, based on the harvesting of wild *I. bauense* as a source of protein supplement naturally may not be practical, and conservation of the species for their ecological roles may thus be preferred.

Conservation of threatened species that is also important to indigenous communities requires substantial ecological and biological information to reconcile the needs. Several other species of freshwater crabs are important as a food supplement to indigenous communities in Sarawak, including *Isolapotamon nimboni* and *Geosesarma katibas* by the Iban community, and *Isolapotamon bauense* by the Bidayuh community (Grinang 2016). Unlike the former two species, threats to *I. bauense* may be more severe due to it localised distributional range and small population size (Grinang *et al*. 2016). Habitat loss/degradation, water pollution, and over-exploitation are the major threats to freshwater crabs globally (Cumberlidge et al. 2009). While impacts of habitat loss and water pollution are easy to identify, over-exploitation of *I. bauense* by indigenous communities needs to be managed wisely. Effects of resources utilisation by indigenous communities have not been well documented, partly due to their relatively infrequent consumption rates. Many studies on nutrient contents had been focused on commercial species of marine crabs (Skonberg & Perkins 2002; Küçükgülmez *et al*. 2006). The present findings seem to be the first in this region that examine nutrient contents of freshwater crabs to provide fundamental knowledge on the nutritional value of crab meat, which can indirectly contribute to sustainable utilisation of the resource and conservation of the species.

## CONCLUSION

Ecological studies of freshwater crabs are crucial for providing information for species conservation and habitat protection. Also, conflicts between conservation programme and utilisation of the resources by indigenous communities need to be managed wisely, which requires substantial information on nutritional value, knowledge of ethnozoology and any related topics. This study demonstrates that freshwater crabs, such as *Isolapotamon bauense,* contain a significant amount of nutrients, but harvesting the species is not practical due to its low abundance of wild populations.
